# Global compositional and functional states of the human gut microbiome in health and disease

**DOI:** 10.1101/gr.278637.123

**Published:** 2024-06

**Authors:** Sunjae Lee, Theo Portlock, Emmanuelle Le Chatelier, Fernando Garcia-Guevara, Frederick Clasen, Florian Plaza Oñate, Nicolas Pons, Neelu Begum, Azadeh Harzandi, Ceri Proffitt, Dorines Rosario, Stefania Vaga, Junseok Park, Kalle von Feilitzen, Fredric Johansson, Cheng Zhang, Lindsey A. Edwards, Vincent Lombard, Franck Gauthier, Claire J. Steves, David Gomez-Cabrero, Bernard Henrissat, Doheon Lee, Lars Engstrand, Debbie L. Shawcross, Gordon Proctor, Mathieu Almeida, Jens Nielsen, Adil Mardinoglu, David L. Moyes, Stanislav Dusko Ehrlich, Mathias Uhlen, Saeed Shoaie

**Affiliations:** 1 Centre for Host-Microbiome Interactions, Faculty of Dentistry, Oral & Craniofacial Sciences, King's College London, SE1 9RT, United Kingdom;; 2 School of Life Sciences, Gwangju Institute of Science and Technology (GIST), 61005, Gwangju, Republic of Korea;; 3 Science for Life Laboratory, KTH–Royal Institute of Technology, Stockholm, SE-171 21, Sweden;; 4 University Paris-Saclay, INRAE, MetaGenoPolis, 78350 Jouy-en-Josas, France;; 5 Department of Bio and Brain Engineering, KAIST, Yuseong-gu, Daejeon 305-701, Republic of Korea;; 6 Institute of Liver Studies, Department of Inflammation Biology, School of Immunology and Microbial Sciences, King's College London, London SE5 9NU, United Kingdom;; 7 INRAE, USC1408 Architecture et Fonction des Macromolécules Biologiques (AFMB), Marseille 13288, France;; 8 Architecture et Fonction des Macromolécules Biologiques (AFMB), CNRS, Aix-Marseille University, Marseille 13288, France;; 9 Department of Twin Research & Genetic Epidemiology, King's College London, London WC2R 2LS, United Kingdom;; 10 Translational Bioinformatics Unit, Navarrabiomed, Universidad Pública de Navarra (UPNA), IdiSNA, 31008 Pamplona, Spain;; 11 Biological and Environmental Science and Engineering Division, King Abdullah University of Science and Technology (KAUST), Thuwal 23955-6900, Saudi Arabia;; 12 Department of Biological Sciences, King Abdulaziz University, Jeddah 21589, Saudi Arabia;; 13 Department of Biotechnology and Biomedicine, Technical University of Denmark, DK-2800 Lyngby, Denmark;; 14 Centre for Translational Microbiome Research (CTMR), Department of Microbiology, Tumour and Cell Biology, Karolinska Institutet, 171 65 Stockholm, Sweden;; 15 Department of Biology and Biological Engineering, Chalmers University of Technology, SE-412 96 Gothenburg, Sweden;; 16 BioInnovation Institute, DK-2200 Copenhagen N, Denmark;; 17 Department of Clinical and Movement Neurosciences, University College London, London NW3 2PF, United Kingdom

## Abstract

The human gut microbiota is of increasing interest, with metagenomics a key tool for analyzing bacterial diversity and functionality in health and disease. Despite increasing efforts to expand microbial gene catalogs and an increasing number of metagenome-assembled genomes, there have been few pan-metagenomic association studies and in-depth functional analyses across different geographies and diseases. Here, we explored 6014 human gut metagenome samples across 19 countries and 23 diseases by performing compositional, functional cluster, and integrative analyses. Using interpreted machine learning classification models and statistical methods, we identified *Fusobacterium nucleatum* and *Anaerostipes hadrus* with the highest frequencies, enriched and depleted, respectively, across different disease cohorts. Distinct functional distributions were observed in the gut microbiomes of both westernized and nonwesternized populations. These compositional and functional analyses are presented in the open-access Human Gut Microbiome Atlas, allowing for the exploration of the richness, disease, and regional signatures of the gut microbiota across different cohorts.

Metagenomic studies have enabled a deeper understanding of the functional potential and taxonomic composition of the microbiome and its implications in identifying health and disease signatures across different body sites and geographic regions (Lozupone et al. 2012; David et al. 2014; Sommer et al. 2017). The large-scale integration of microbiome functional changes and their associations with clinical data could provide new insights into their impact on host physiology and disease pathophysiology, as well as new microbiome-based treatments and therapies (Lozupone et al. 2012; David et al. 2014). Recently, several studies have focused on the discovery of new uncultured microbes through the generation of metagenome species (Nielsen et al. 2014; Almeida et al. 2019; Nayfach et al. 2019; Plaza Oñate et al. 2019), whereas others have focused on the investigation of alterations in microbiome composition owing to disease, geographical location, and interventions in the gut microbiome (Jalanka-Tuovinen et al. 2011; David et al. 2014; Mehta et al. 2018; Pasolli et al. 2019).

The key to advancing our understanding of the critical role of the microbiome in health and disease is access to data from a wide range of studies and cohorts. Public resource collection and processing of microbiome data are essential, contributing to the laborious and necessary task of standardizing and making this accumulated information accessible. Some have particularly focused on the human gut microbiome: gutMDisorder (Cheng et al. 2020), GIMICA (Tang et al. 2021), Disbiome (Janssens et al. 2018), and GMrepo (Wu et al. 2020). However, there is a lack of integrative functional and compositional analyses across cohorts and regions to provide a mechanistic understanding of the microbiome and identify biomarkers. In this study, we integrated publicly available data from a wide range of studies across different countries from both healthy and diseased individuals. To overcome the current limitations of meta-analyses of microbiome studies, we used a machine learning approach to extract microbial features from different diseases. We calculated the enrichment of microbial species for both disease and geographical regions and performed Shapley additive explanations (SHAP) interpretations on random forest classification models to identify biomarkers of disease associated with metagenomic species pan-genomes (MSPs). Additionally, we present an open-access Human Gut Microbiome Atlas (HGMA) (https://www.microbiomeatlas.org) that allows researchers to explore an integrative analysis of compositional, functional, richness, disease, and regional signatures of the gut microbiota across 19 geographical regions and 23 diseases.

## Results

### The HGMA: a pan-metagenomic study of compositional and functional changes of the human gut microbiome

**Figure 1.**
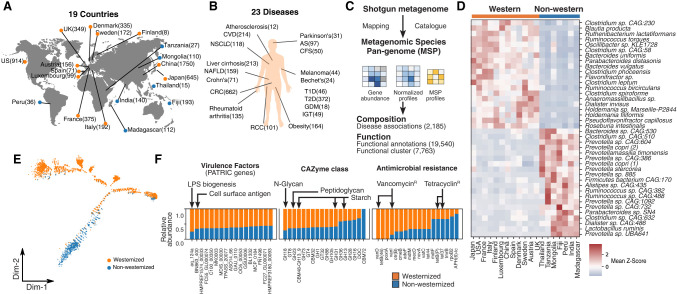
Characterization of the global gut microbiome in health and disease. Pan-metagenomics association studies of health and disease. Corresponding data sets were publicly shared as a resource: the Human Gut Microbiome Atlas (HGMA). (*A*) The geographical distribution of the data sets used in this study (the number of the samples is shown in parentheses). (*B*) Disease data sets of shotgun metagenomics used in this study. (*C*) The workflow of the metagenomic species pan-genome (MSP) quantification together with functional characterization. We first constructed 1989 MSPs for gut microbiome by MSPminer based on co-abundant gene profiles, which give clues to identify gene cluster markers likely belonging to the same species. Next, all the short reads aligned to the IGC2 catalog and, subsequently, gene abundances were profiled, downsized, and normalized. Based on co-abundant gene markers from the given MSP, mean signals were used to estimate species abundance profiles. In total, 6014 shotgun metagenome samples were aligned against the gene catalog of the human gut microbiome and quantified at the level of MSP. (*D*) Heatmap showing the top 20 significantly overrepresented MSPs between western and nonwestern cohorts colored by mean species *Z-*score for each country against all countries. (*E*) Monocle ordination of the gut microbiome. Individual samples from nonwestern and western countries were colored blue and orange, respectively. (*F*) Difference in gene content between western and nonwestern enriched species. Those species gene content was annotated by those that were CAZymes, antimicrobial-resistance (AMR) genes, and virulence factors (PATRIC database) and summed across all species. Total number of each gene was normalized and plotted as a stacked bar plot to show regional overrepresentation (Methods).

We analyzed 6014 publicly available shotgun metagenomic stool samples to create a public resource for investigating the microbiome across diverse settings. A total of 6014 samples with at least 10 million high-quality sequencing reads were selected from healthy and diseased cohorts from 19 different countries across five continents (Fig. 1A,B; Supplemental Table S1). We included metagenomic samples of normal subjects in nonwesternized countries for comparison of the differences between westernized and nonwesternized regions and later with disease signals; however, disease samples from nonwesternized regions were very limited and thus were not included in this study. We normalized all metagenomic sample abundances to enable comparative analysis across cohorts (Methods). Using these samples, we created the HGMA by quantitative analysis of shotgun metagenomics based on microbial genomes assembled using MSPs (Fig. 1C; Supplemental Fig. S1A). We generated gene counts using the IGC2 10.4 million gene catalog from all raw metagenome data and, after normalization of gene counts, profiled MSP abundances for all samples based on the co-abundant gene markers of given MSPs (Wen et al. 2017). We further characterized the functions and phenotypes of the identified MSPs in seven categories: KEGG orthologs (KOs) (Kanehisa et al. 2004), protein families (Pfam) (Bateman et al. 2004), carbohydrate-active enzymes (CAZymes) (Terrapon et al. 2017), antimicrobial resistance (AMR) (Ruppé et al. 2019), microbial phenotype (Mukherjee et al. 2021), virulence factors (Mao et al. 2015), and biosynthetic gene clusters (BGCs) (Blin et al. 2017). We identified 7763 co-conserved functional clusters across species (Methods). All these data are freely available in the HGMA without restrictions in the public open-access database (https://www.microbiomeatlas.org).

Using all cohorts, we determined the geographical distribution of the gut microbiome. Both *Clostridium* and *Bacteroides* were found to have higher mean relative abundance within western countries, whereas *Prevotella* species had a higher mean relative abundance within nonwestern countries (Fig. 1D; Supplemental Table S2), in accordance with previous studies (Yatsunenko et al. 2012). We applied an unsupervised clustering method, Monocle, to MSP abundance profiles of all samples (Methods) (Trapnell et al. 2014; Qiu et al. 2017) and observed that there were two distinct ordinations of nonwesternized and European samples of subjects connected by a mixture of western/nonwestern samples belonging to China or Japan and to the United States (Fig. 1E; Supplemental Fig. S1B). Based on a comparative analysis across different regions, we identified 742 MSPs specifically enriched in certain regions (Methods) (Supplemental Table S3). Functional annotation analysis across geographical clusters revealed enrichment of CAZymes for degrading N-glycans, food carbohydrates of animal origin, and storage carbohydrates in westernized populations, in which AMR and virulence factors were also more prevalent (Fig. 1F; Supplemental Table S4). A comparison of the functions of region-enriched MSPs in westernized countries revealed that genes encoding vancomycin resistance and lipopolysaccharide (LPS) biogenesis are overrepresented. An overrepresentation of genes encoding complex polysaccharide-binding proteins mostly belonging to the *Prevotella* genus was found in the nonwesternized cohorts (Prasoodanan et al. 2021), and we identified that the cluster for vancomycin resistance was enriched in the westernized population, whereas the tetracycline-resistance cluster was enriched in the nonwesternized population.

### Pan-metagenomics association study across 23 diseases

**Figure 2.**
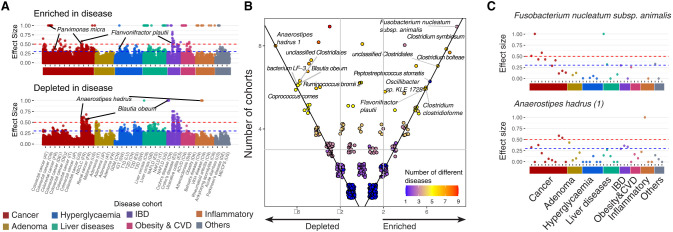
Pan-metagenomics association studies (Pan-MGAS) of 43 cohorts from 23 different diseases and 14 countries (n = 2185). (*A*) We identified significantly enriched (*top*) and depleted (*bottom*) species of cohorts based on the effect sizes (ESs) of Wilcoxon rank-sum one-sided tests (ES ≥ 0.3; i.e., each dot represents the ES of an MSP in each disease data set); the complete list of values is provided in Supplemental Table S5. The blue dotted line indicates ES = 0.3; the red dotted line indicates ES = 0.5; and each dot in the plot represents one MSP within one disease cohort. (*B*) Scatter plots of the frequency of the significantly enriched/depleted cohorts of all MSPs (ES > 0.3): Each point represents an MSP; all values in the plot are integers; and jitter was added to remove overlapping points. The *y*-axis displays the total frequency of enriched/depleted cohorts (number of enriched cohorts + number of depleted cohorts), and the *x*-axis displays the subtracted frequency between enriched cohorts and depleted cohorts (number of enriched cohorts − number of depleted cohorts). Point coloring is based on the number of different diseases for which an MSP had an ES above 0.3. Commonly enriched/depleted species among cohorts were identified when total frequency ≥ 3 and absolute subtracted frequency ≥ 2. (*C*) Species found depleted (*Anaerostipes hadrus*) and enriched (*Fusobacterium nucleatum subsp. animalis*) in most disease cohorts. The blue dotted line indicates ES = 0.3; the red dotted line indicates ES = 0.5; and each dot in the plot represents one MSP within one disease cohort. Acronyms are as follows: (ACVD) acute coronary cardiovascular disease, (Ob) obesity, (CRC) colorectal cancer, (NSCLC) non-small-cell lung cancer, (RCC) renal cell carcinoma, (GDM) gestational diabetes mellitus, (T1D) type 1 diabetes, (T2D) type 2 diabetes, (LC) liver cirrhosis, (NAFLD) nonalcoholic fatty acid liver, (UC) ulcerative colitis, (CD) Crohn's disease, (BD) Behçet's disease, (RA) rheumatoid arthritis, (SPA) ankylosing spondylitis, (ME/CFS) myalgic encephalomyelitis/chronic fatigue syndrome, and (PD) Parkinson's disease.

We performed a pan-metagenomics association study (Pan-MGAS) of multiple disease cohorts (23 diseases across 43 cohorts from 14 countries) to distinguish between diseased versus healthy microbiomes within multiple cohorts. We reported the enriched and depleted species within the different disease cohorts compared with healthy samples from the same country, by determining the effect size and using the magnitude of enrichment/depletion of given species in abundance (greater than the medium effect size, 0.3; Methods) (Fig. 2A; Supplemental Tables S5, S6; Supplemental Fig. S2A,B). Some cohorts showed depletion of multiple species, notably in cancer (non-small-cell lung cancer [NSCLC] from France, renal cell carcinoma [RCC] from France, and adenoma from Italy) (Fig. 2A). Conversely, some diseases have several enriched species, as observed in most colorectal cancer (CRC) cohorts.

To explore the connection between enriched species in both healthy western and nonwestern populations with disease, we investigated the effect size values for those enriched species across disease cohorts (Supplemental Fig. S2C). We found that effect sizes center around zero in both groups, suggesting most of these species exhibit little change in response to disease. This observation does not, however, rule out the possibility that there is a connection between region and disease that warrants future analysis to explore.

In our Pan-MGAS analysis, some species were either enriched or depleted across multiple cohorts, regardless of geographical differences. For example, *Anaerostipes hadrus* and *Coprococcus comes*, which have been associated with healthy individuals, are among the most frequently depleted species found in at least six different disease cohorts (Fig. 2B; Supplemental Fig. S3). The two species have been described as butyrate producers and are the dominant species isolated from the healthy human colon (Holdeman and Moore 1974; Louis and Flint 2009; Allen-Vercoe et al. 2012).

Between the species found enriched in at least six different cohorts, we find *Fusobacterium nucleatum*, *Clostridium bolteae*, *Clostridium clostridioforme*, *Clostridium symbiosum*, *Peptostreptococcus stomatis*, *Flavonifractor plautii*, *Parvimonas micra*, among others (Fig. 2C; Supplemental Fig. S3). Several of them have also been isolated from oral samples (*F. nucleatum* [Socransky et al. 1998], *P. stomatis* [Downes and Wade 2006], *P. micra* [Rôças and Siqueira 2008]), and some have been identified in infections, including bacteremia (*C. bolteae* [Finegold et al. 2005], *C. clostridioforme* [Finegold et al. 2005], *P. micra* [Löwenmark et al. 2020]). Along with *F. nucleatum* and *C. symbiosum*, which are enriched in western countries and are associated with CRC (Elsayed and Zhang 2004; Castellarin et al. 2012; Kostic et al. 2012), we also identified *P. micra* to be enriched in multiple cohorts of CRC, and *P. stomatis* enriched several times in solid tumor cohorts (Supplemental Table S6; Supplemental Fig. S3).

### Disease-enriched functional clusters show distinct links to gut microbiome dysbiosis

**Figure 3.**
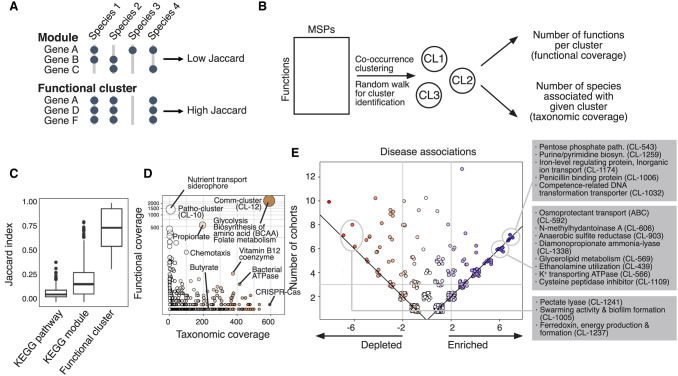
Analysis of functional clusters of the human gut microbiome. For the functional characterization of human gut MSPs, we annotated respective genes with 19,540 features of microbial function/phenotype databases and identified 7763 functional clusters better representing the microbiome. (*A*) Identification of functional clusters based on co-conserved molecular and biological functions across species. Unlike the manually curated module database, we identified functional clusters based on high co-conservation across species using the unsupervised clustering method. (*B*) The overall scheme of identification of functional clusters and checking functional coverage (cluster size) and taxonomic coverage (number of enriched species). (*C*) We found that among different sources of microbial functional annotations (e.g., KEGG module and pathway), co-conservation of molecular and biological functions across different species was substantially low (Jaccard index < 0.5). (*D*) Functional clusters identified by unsupervised community detection. The *y*-axis displays the number of genes within the functional cluster (i.e., functional coverage), and the *x*-axis displays the number of MSPs possessing >70% of the clusters’ genes (i.e., taxonomic coverage). (*E*) Functional clusters projected on enriched/depleted MSPs across disease cohorts. The scatter plot displays the frequency of functional clusters significantly associated with the enriched/depleted species (hypergeometric test *P*-value < 0.0001) in disease cohorts. Each point represents a gene cluster; all values in the plot are integers; and jitter was added to remove overlapping points. The *y*-axis shows the total frequency of cohorts in which a functional cluster was found significantly associated with enriched/depleted species. The *x*-axis shows the difference in the number of cohorts in which a function was found enriched minus the frequency it was found depleted. Point colors changed from red (*left*) to blue (*right*) according to *x*-axis values. Common enriched/depleted functional gene clusters among cohorts were identified when total frequency ≥ 3 and absolute subtracted frequency ≥ 2.

To analyze the functional content in the MSP from the human microbiome, we applied an unsupervised clustering approach to the annotated functions (Methods) (Fig. 3A,B; Supplemental Fig. S4A,B). This analysis provided a better representation of microbial functions than single annotations or known pathway definitions (e.g., KEGG) (Fig. 3C). We identified 7763 functional clusters and 6297 singletons using the community detection algorithm (Supplemental Fig. S4C; Supplemental Table S7). For example, AMR and secondary biosynthetic genes were found to be singletons that were not co-conserved with other functional genes. After excluding singletons and unreliable functional clusters detected in fewer than three species, 591 representative clusters of microbial functions were retained. One of the two largest clusters (CL-12, named “*comm-cluster*” hereafter) (see Supplemental Table S7) was overrepresented among many commensal species, whereas the other (CL-10, named “*patho-cluster*”) was enriched in a few pathobionts, such as *Klebsiella* spp., *Enterobacter* spp., and *Eschrichia coli*. The *comm-cluster* was enriched with genes involved in the biosynthesis of amino acids. In contrast, the *patho-cluster* was enriched in functions associated with uptake of several substrates. These include siderophores, amino acids, and vitamin transport, thus improving competitive fitness against commensal bacteria. We also found other functionally enriched clusters, such as the butyrate metabolism cluster, propionate metabolism cluster, and CRISPR-Cas system cluster (Fig. 3D); a number of these were correlated with phylum-level taxonomy (Supplemental Table S7).

Next, we extracted the enriched/depleted species in each cohort and recovered the functional clusters associated with these species (hypergeometric tests, *P*-value < 10^−4^) (Fig. 3E). We found several functional clusters commonly associated with the enriched species in the disease. Among these, we found that CL-1006 is related to antibiotic resistance; CL-1032, a competence-related DNA transformation transport, could provide an advantage by improving the integration of new functions into the genome; or clusters related to metabolic pathways that could contribute indirectly to pathogenicities, such as the pentose phosphate pathway (Rytter et al. 2021) or ethanolamine utilization (Garsin 2010). Among the most frequent functional clusters that accompany the depleted species in disease, we found the CL-12 comm-cluster and other clusters with functions related to pectate degradation and biofilm formation (Fig. 3E), all of which were related to the normal function of the healthy microbiota.

### Global view of gut MSPs

To obtain a holistic view of human gut MSPs, we generated a phylogenetic tree displaying the taxonomic resolution of disease- and region-enriched species and estimated proportionality between MSP pairs (Methods) (Supplemental Fig. S5A). Most MSPs are present in both the western and nonwestern regions. Although some were enriched in one of the two regions, we could not identify any apparent phylogenetic pattern. When looking at the enrichment/depletion across the different cohorts, the *Streptococcus* genus showed particularly interesting features: Members within this genus were found to be enriched in some cohorts while being depleted in others. For example, three different species within the genus (*Streptococcus anginosus*, *Streptococcus parasanguinis*, and *Streptococcus vestibularis*) were enriched in two distinct liver disease cohorts, whereas *Streptococcus salivarius* and *Streptococcus sanguinis* were depleted in cancer cohorts (Supplemental Fig. S5B; Supplemental Table S6).

In addition, we observed proportionality between the MSPs (Supplemental Fig. S5C). A high proportionality value between a pair of MSPs suggests that they tend to increase or decrease simultaneously. Most MSPs with the highest proportionality values belonged to the same genus. Only a small subset of MSPs with proportionality values above the selected threshold was found. Many of the MSP pairs we found were inhabitants of the oral cavity, and the *Streptococcus* genus stood out again. Bacterial infections of the *Streptococcus* genus play a central role from a clinical perspective (Krzyściak et al. 2013; Marzhoseyni et al. 2022).

### A random forest classification model can identify biomarkers of disease from MSPs

**Figure 4.**
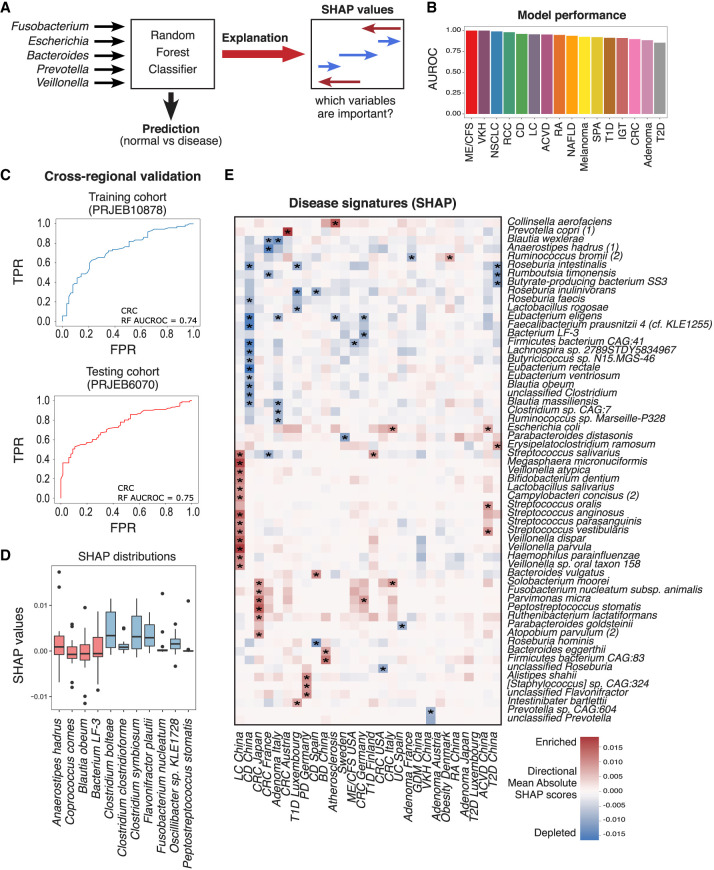
Random forest (RF) models trained on multiple cohorts to discriminate between disease and healthy controls. (*A*) Schematic of RF classification method. (*B*) AUROC scores for each disease RF classification model. (*C*) AUROC curves of an inter (*top*) and intra (*bottom*) cohort validation for a RF model that predicts CRC. (*D*) Box plot of directional mean absolute SHAP scores for all disease predictive models. Red and blue boxes represent species that were depleted/enriched using effect size calculation. (*E*) Clustered heatmap (dendrogram omitted) of the most important species for prediction of 16 diseases by RF classification as calculated by directional mean SHAP score (rows contain at least one species with directional mean SHAP score above 0.0125 in any of the diseases; Methods). Positive values indicate that higher relative abundance is more likely to classify the disease versus healthy samples. Negative values indicate that lower relative abundance is more likely to classify the disease versus healthy samples. The *right* color bar indicates mean species bias for enrichment or depletion in all diseases. Acronyms are as follows: (CRC) colorectal cancer, (NSCLC) non-small-cell lung cancer, (RCC) renal cell carcinoma, (T1D) type 1 diabetes, (T2D) type 2 diabetes, (LC) liver cirrhosis, (NAFLD) nonalcoholic fatty acid liver, (CD) Crohn's disease, (RA) rheumatoid arthritis, (SPA) ankylosing spondylitis, (ME_CFS) myalgic encephalomyelitis/chronic fatigue syndrome, (IGT) impaired glucose tolerance, and (VKH) Vogt–Koyanagi–Harada.

To identify disease biomarkers, we implemented feature-selection-based random forest classifier models trained using the MSPs constructed from each cohort on the HGMA for each disease cohort that had matched healthy controls (Fig. 4A). These models were able to distinguish between the diseased and control groups with variable discriminatory performances. Prediction performance was evaluated using the area under the ROC curve (AUROC) metric (Fig. 4B). As a consequence of the disproportionate sample numbers for each disease, we recognize that overfitting was a possibility during the analysis for those diseases with low sample numbers. Nonetheless, the models with the highest predictive capabilities were those for myalgic encephalomyelitis/chronic fatigue syndrome (ME/CFS), Vogt–Koyanagi–Harada (VKH), and Crohn's disease (CD).

The generalization of these models was assessed with inter-study cross-validation, which demonstrated that a model trained on the CRC training cohort (Yu et al. 2017) was able to maintain the predictive power of disease classification when applied to the CRC test cohort (AUROC = 0.74) (Fig. 4C; Zeller et al. 2014). Additional validation of the importance of randomly selected healthy samples was performed by combining 30 random groups of 40 healthy samples with 40 random CRC samples and repeating the cross-validation. The AUROC of predicting the test cohort was 0.75 ± 0.04, showing conservation of predictive capabilities.

The interpretable machine learning framework SHAP was used to identify disease-specific gut microbiome features (Štrumbelj and Kononenko 2014). SHAP is a state-of-the-art framework that has recently been used to explain gut microbiome classification models (Manor and Borenstein 2017; Bar et al. 2020). By interpreting the disease classification models using directional mean absolute SHAP values, the importance of metagenomic species as biomarkers for 16 diseases in the HGMA was measured (Supplemental Table S8).

When comparing SHAP score-calculated biomarkers to effect size biomarkers for all diseases, several key species were shared (Fig. 4D). The highest directional mean SHAP scoring species for the CRC predictive model were *F. nucleatum*, *P. micra*, *Solobacterium moorei*, and *S. parasanguinis*, all of which are known species biomarkers (Li et al. 2016; Löwenmark et al. 2020; Rebersek 2021).

Of note, an increase in the abundance of commensal oral bacteria, including *Haemophilus parainfluenzae*, *Veillonella dispar*, *Veillonella atypica*, and *Veillonella parvula*, was shown to be highly important in predicting liver cirrhosis but not nonalcoholic fatty liver disease (NAFLD), as found previously (Patel et al. 2022), and was found to be enriched in multiple cohorts regardless of region (Fig. 4E). In the NAFLD model, an increase in the abundance of *S. parasanguinis* was the most important factor in predicting the disease. This species was found to be enriched across multiple cohorts of HGMA and is part of a cluster of oral commensal species previously shown to be biomarkers of the disease (Behary et al. 2021). NAFLD also shares biomarkers with T1D, including *A. hadrus* and *Eubacterium hallii*. These two diseases have previously been shown to be metabolically associated (Cusi et al. 2017).

There were some diseases in which the most highly important species for disease prediction were depleted (such as CD). Additionally, there were several shared disease-predictive species, such as *S. parasanguinis* and *Dorea longicatena*, with their presence and absence characterizing a general dysbiotic state (Fig. 4E; Supplemental Fig. S6).

## Discussion

One of the most pressing requirements to allow us to fully realize the potential of the wealth of data we can now generate around the microbiome to our understanding of disease is integrated resources for assessing and analyzing data from a wide range of different studies. Here, we performed a comprehensive integrative analysis of global and temporal gut microbiomes and developed an open-access HGMA portal (https://www.microbiomeatlas.org) to enable browsing these data sets. This resource allows for the integration of several studies linking species to disease, region, and function. It also presents a means for phylogenetically contextualizing gene and species enrichment, as well as identifying common features. Notably, the difference in origin (western/nonwestern) is reflected in the gut microbial composition, with species/genes being over- or underrepresented in the different regions. Importantly, some species and functions were enriched or depleted across multiple diseases and studies with a number of these species being important predictors of diseases.

Increasing numbers of shotgun metagenomic studies have been conducted in the past decade. However, because of inconsistencies in bioinformatic pipelines and microbiome references used along with the difficulties in correcting confounding factors owing to lack of clinical metadata, a proper meta-analysis of these shotgun metagenomic studies has to date not been performed. Many recent studies have now introduced machine learning approaches to overcome confounding effects and large per-study variations by cross-validations (Wirbel et al. 2021). In this study, we applied a standardized bioinformatics pipeline with machine learning approaches to overcome the challenges in meta-analysis of shotgun metagenomic studies.

Within microbiome research, there are limitations owing to batch effects, confounding factors (e.g., age, sex, or ethnicity), DNA extraction protocols, and use of differential abundance analysis tests. Here, we performed PERMANOVA tests for country-matched controls (that did not have matched controls in the study) and found there is no significant effect on microbiome composition (Supplemental Table S9). Moreover, we also applied PERMANOVA to calculate the association of age, BMI, and gender and found there is a limited effect on the microbiome composition (Supplemental Table S10). Some approaches exist for removing batch effects in microbiome data sets (Ling et al. 2022; Ma et al. 2022); however, they assume that all confounding factors are known, which can be challenging when public data sets are used that do not provide metadata. This can limit the power of batch effect correction techniques, potentially leading to reduced statistical power or confounding with batch-introduced variation. This stresses the need for careful experimental planning and the importance of every research group to document confounding variables in their public data sets (Soneson et al. 2014).

In addition, effect size estimates based on nonparametric tests might be different from the linear modeling after data transformations, such as centered log ratio (CLR) and log-transformations. Differences between identified species using distinct differential abundance methods have been documented before (Nearing et al. 2022; Yang and Chen 2022), although no consensus exists on what is the best approach. Therefore, we applied MMUPHin (Ma et al. 2022) for batch effect correction and regression analysis with MaAsLin 2 (Mallick et al. 2021) and aggregated the results using a fixed/mixed effect model with default parameters. These outputs are in the HGMA (Supplemental Table S11). We assumed that cohorts of the same disease type might share common effect sizes for disease-associated microbes, akin to fixed-effect models. However, such assumptions might need further validation in independent data sets.

Confirming previous observations (Yatsunenko et al. 2012; Pasolli et al. 2019), we described the regional specificity of the gut microbiome, which needs to be considered before using the gut microbiome for patient stratification or designing intervention studies. In addition, we found that there were distinctions in functions enriched in westernized and nonwesternized countries, including resistance to vancomycin and tetracycline, respectively. Interestingly, we found some difficulty in defining geographic regions into western versus nonwestern countries or into industrialized versus nonindustrialized countries. Thus, regional specificity needs further investigation of lifestyle or diet factors that can drive this regional dichotomy.

The physiological changes caused by the disease might partly explain why some diseases have a pronounced compositional imbalance whereas others do not. Diseases affecting the bowel show a high effect size for many species, whereas diseases affecting other body parts tend to produce smaller imbalances. Other factors might also be involved in the magnitude of the imbalance, for example, changes in diet (Shen et al. 2014; Riaz Rajoka et al. 2017) or the use of drugs for treating the disease (Le Bastard et al. 2018; Vich Vila et al. 2020; Weersma et al. 2020).

Notably, we observed that some of the more frequently depleted species in disease were butyrate producers. Butyrate has been associated with beneficial effects in the colon (inhibition of inflammation, reinforcement of the epithelial barrier, decreased oxidative stress) (Hamer et al. 2008). In addition, butyrate-producing species are also depleted in our models of CD presented here, suggesting that depletion of health-driving species is as significant as enrichment of disease-driving species in disease status. Conversely, some enriched species may induce disease pathology by driving new infections, potentiating disease symptoms, or even weakening immune responses. For example, some reports suggest *F. nucleatum* promotes CRC development and metastasis (Casasanta et al. 2020; Chen et al. 2020). We found a similar link using the SHAP-interpreted random forest predictive model. Others report that *F. plautii*, a species enriched in six cohorts in the HGMA, suppresses Th2 immune responses in mice (Ogita et al. 2020), suggesting that this species exerts a similar effect in humans. The Pan-MGAS we present here is dominated by CRC studies because of the greater number of these data sets available. As future studies include more countries and diseases, our analyses will be updated and balance out this bias.

All the species in the present study were derived from metagenomic gut samples; however notably, many of the species identified in our analyses as either enriched or depleted in disease states are not exclusively found in the gut but also present in the oral cavity. This is particularly true for representatives of the *Streptococcus* genus. Many of the streptococcal species identified here are members of the *viridans* group *Streptococci*, a diverse group that has members associated with disease and polymicrobial infection (e.g., *S. anginosus*, associated with liver and soft tissue abscesses) (Conte et al. 2020), as well as members that have been proposed for use as probiotics (e.g., *S. salivarius*) (Shen et al. 2014; Riaz Rajoka et al. 2017). We recently found that the translocation of oral bacteria to the gut may lead to systemic inflammation during disease pathogenesis, including liver cirrhosis, and rifaximin treatment may prevent this oralization, thereby improving disease symptoms, including hepatic encephalopathy (Patel et al. 2022).

The projection of functions associated with enriched/depleted species in disease supports observations made using species alone. Functions commonly enriched in diseases potentialy provide their carriers with increased ecological fitness, meaning that they have a better chance of thriving in altered conditions, playing indirect roles in disease pathology, for example, by utilizing additional carbon sources (e.g., CL543-pentose phosphate pathway [Rytter et al. 2021], ethanolamine [Garsin 2010]) or increasing their ability to survive environmental stresses (CL-592 osmoprotectant cluster) (Riaz Rajoka et al. 2017). However, enrichment of these functions does not mean that they are exclusive to pathogenic organisms. For example, although anaerobic sulfite-reducing activity is often used as a marker for food contamination (Doyle et al. 2018), it is also present in several nonpathogenic bacteria. Conversely, functions depleted in different diseases may also play an active role in health maintenance. For example, pectic substances can inhibit gut inflammation and relieve inflammatory bowel disease symptoms (Doyle et al. 2018).

Finally, the integration of metagenomic data from many studies spanning five continents provides a valuable knowledge resource for researchers investigating the impact of the microbiome on individual health parameters. This open-access atlas will be updated routinely with the new publicly available gut metagenomics data, including the recently announced One Million Microbiome Project, to provide comprehensive open-access metagenomics data from multiple research centers. Therefore, an in-depth analysis of the impact of the gut microbiome on health and disease will be used to facilitate future studies to reveal the critical role of the gut microbiome in maintaining human health.

## Methods

### Metagenomics species pan-genome creation

The 1601 metagenomic samples used to build the Integrated Gene Catalog of the human gut microbiome (IGC2) were downloaded from the European Nucleotide Archive (ENA; https://www.ebi.ac.uk/ena/browser/home) (Supplemental Fig. S1A; Li et al. 2014). Using the Meteor software suite (Pons et al. 2010; see https://forgemia.inra.fr/metagenopolis/meteor), reads from each sample were mapped against the IGC2 catalog, and a raw gene abundance table was generated. This table was submitted to MSPminer (Plaza Oñate et al. 2019), which reconstituted 1989 metagenomic-species pan-genomes (MSPs). MSPs are gene clusters that most likely belong to the same species (with a genome average nucleotide identity ≥ 95%), based on the hypothesis introduced by Nielsen et al. (2014) that genes belonging to the same species should be co-abundant across multiple metagenomic samples (Supplemental Materials). In this study, MSPs were used to identify species-specific core genes that allow for high-sensitivity and high-specificity taxonomic profiling. The remaining genes that were part of the pan-genome of the species were also used to assess the functional potential of microbial species or to study intra-species gene content variability. Quality control of each MSP was manually performed by visualizing heatmaps representative of the normalized gene abundance profiles. In addition, MSP completeness and contamination were assessed by searching for 40 universal single-copy marker genes (Sunagawa et al. 2013) and by checking taxonomic homogeneity.

### MSP taxonomic annotation with phylogenetic tree

MSP taxonomic annotation was performed by aligning all core and accessory genes against *nt* and NCBI WGS (version of September 2018 restricted to the taxa bacteria, archaea, fungi, viruses, and blastocystis) using BLASTN (version 2.7.1, task = megablast, word_size = 16) (Altschul et al. 1997). The 20 best hits for each gene were retained. A species-level assignment was given if >50% of the genes matched the RefSeq reference genome of a given species, with a mean identity of ≥95% and mean gene length coverage of ≥90%. The remaining MSPs were assigned to a higher taxonomic level (genus to superkingdom) if >50% of their genes had the same annotation.

Forty universal phylogenetic marker genes were extracted from MSPs using MOCAT (Kultima et al. 2012). MSPs with fewer than five markers were excluded. The markers were then aligned separately using MUSCLE (Edgar 2004). Forty alignments were merged and trimmed using trimAl (Capella-Gutiérrez et al. 2009). Finally, the phylogenetic tree was computed using FastTreeMP (Price et al. 2010) and visualized using iTOL (Letunic and Bork 2021). Phylogenetic placement was used to improve and correct taxonomic annotation. Phylogenetic data, species labels, and phylum coloring can be accessed from the INRAE data portal (https://data.inrae.fr/dataset.xhtml?persistentId=doi:10.15454/FLANUP), with annotations for enriched species found at GitHub (https://github.com/sysbiomelab/ATLAS).

### Functional annotation of the gut gene catalog and MSP

The IGC2 catalog was annotated for antibiotic-resistance determinants (ARDs) described in the Mustard database (v1.0) (Ruppé et al. 2019; http://www.mgps.eu/Mustard/). Protein sequences were aligned against 9462 ARD sequences using BLASTP 2.7.1+ (option –evalue = 10^–5^). Best-hit alignments were filtered for identity ≥95% and bidirectional alignment coverage ≥ 90% (at the query and subject levels), giving a list of ARD candidates belonging to 30 families. Annotation of the CAZymes of the IGC2 catalog was performed by comparing the predicted protein sequences to those in the CAZy database and to hidden Markov models (HMMs) built from each CAZyme family (Terrapon et al. 2017), following a procedure previously described for other metagenomic analyses (Svartström et al. 2017). Proteins in the IGC2 catalog were also annotated to KOs using DIAMOND (version 0.9.22.123) (Buchfink et al. 2015) against the KEGG database (version 82). Best-hit alignments with an e-value ≤ 10^−5^ and bit scores ≥ 60 were considered. Proteins involved in the virulence factors of PATRIC (Gillespie et al. 2011; Mao et al. 2015) were matched against IGC2 (Li et al. 2014) using BLASTP (best identity >50%, e-value < 10^−10^). The phenotypes of MSPs were manually checked and annotated based on the JGI-GOLD phenotype (organism metadata) (Mukherjee et al. 2019). We identified the biosynthetic genes of MSPs using the standalone antiSMASH program with the minimal run option, focusing on core detection modules (version 5) (Blin et al. 2017).

### Quality control/normalization of gene counts and species abundance profiling

We collected 6014 of gut microbiome samples across 19 different countries. To assess the technical biases for the DNA extraction, we checked for all the available extraction protocols (Supplemental Table S12). Only four data sets (8% of total samples) stated that in-house DNA extraction protocols were used, whereas other data sets used standard protocols or commercially available extraction kits (MB Biomedicals, MoBio, Qbiogene, and Qiagen). To test degrees of variability in outputs, we performed PERMANOVA tests and concluded that different DNA extraction protocols do not have a significant effect on microbiome composition (Df = 14, F = 5273, *P*-value = 0.422). We filtered out human reads and then mapped metagenomic data on the IGC2 catalog of the human gut metagenome using METEOR (Pons et al. 2010). Based on the aligned reads, we estimated the abundance of each reference gene in the catalog, normalizing multiple mapped reads by their numbers and summing up normalized counts for a given gene. To reduce the variability by sequencing depths, gene count values were downsized to 10 million reads per sample, and samples with fewer than 10 million mapped reads were excluded from our data set. Normalized gene counts were used to quantify MSP abundance using the R *momr* (*MetaOMineR*) package (Le Chatelier et al. 2013). MSP abundances were estimated by the mean abundance of its 100 “marker” genes (i.e., the genes that correlate the most altogether). If <10% of “marker” genes were seen in a sample, the abundance of the MSPs was set to zero.

### Tracing the diversification of healthy metagenomic samples of different geographies

After quantification and per-million scaling of MSP abundance profiles, we employed trajectory analysis in the R Monocle version 2 package to identify how samples were clustered (Qiu et al. 2017). In short, we selected the species profiles of all normal samples from different geographical origins and reduced the sample profiles into two dimensions using the advanced nonlinear reconstruction algorithm *DDRTree*. Based on the reduced two-dimensional components, we presented how the samples were closely clustered as branches in the scatter plots.

### Identification of region-enriched species and genes from geographically distinct cohorts

The regional enrichment of species was calculated by the *Z*-score for each MSP from the difference between the mean relative abundance of each country and the entire population. By selecting the top 100 overrepresented MSPs in the western and nonwestern groups, two separate cumulative sums of their genes were filtered to obtain more than 90 genes. The genes in each list were mapped against the CAZy, PATRIC, and Mustard databases. Eighteen of the maximum differences between the western and nonwestern gene count lists were calculated and plotted.

### Pan-metagenomics association studies

First, we selected healthy and diseased samples without interventions and redundant measurements (i.e., multiple visits) and performed comparative analyses of the chosen samples (for the number of selected samples, see Supplemental Table S1). We estimated the effect sizes of Wilcoxon rank-sum (one-sided) tests for MSP enrichment and depletion in diseases compared with healthy controls in a given country (Fritz et al. 2012) and identified significantly enriched or depleted species with medium effect sizes (effect size ≥ 0.3). To estimate the effect-size values, the Z-statistics calculated from the *P*-value were divided by the square root of the total number of samples (Fritz et al. 2012). Manhattan plots of Pan-MGAS based on effect sizes were plotted using the R qqman package (Turner 2018). To identify the MSPs frequently enriched or depleted in disease, we counted the number of times each MSP had an effect size above 0.3 in each different disease cohort included in this study.

### Unsupervised clustering of co-conserved functions of gut microbiota

We calculated the Jaccard index among functional annotations to calculate the number of species that shared a pair of functions, which were compared at the annotated term levels not the gene levels. We selected highly shared pairs of functions (Jaccard index ≥ 0.75) and merged them into a functional co-occurrence network using the R igraph package (Csardi and Nepusz 2006). Functional clusters within the network were identified by unsupervised community detection, the short random walk algorithm (*cluster_walktrap* function) (Pons and Latapy 2006; Uhlen et al. 2017), and singleton functions within the network. Among nonsingleton functional clusters, we selected representative functional clusters if the functions of given functional clusters were found in more than three species, thereby excluding functional clusters sparsely annotated over MSPs. MSPs were associated with a functional cluster if the given MSP covered >75% of the functions of the functional cluster (Supplemental Table S7).

### Enrichment of functional clusters in disease cohorts

To project the functional clusters associated with enriched/depleted MSPs within a disease cohort, we applied a hypergeometric test to determine the probability of finding the set of MSPs associated with the functional cluster overlapping with the set of enriched/depleted MSPs within the disease cohort. We only applied the test to those functional clusters with at least 10 associated MSPs and established a *P*-value cutoff of 0.0001. To identify the functional clusters frequently enriched in disease, we counted the number of times each cluster had a *P*-value below the cutoff in each different cohort included in this study.

### Proportionality between MSPs

Proportionality was estimated using the propr R package (Quinn et al. 2017). We used the relative abundance matrix of all samples against the MSP as the input. Only MSPs with relative abundance values above zero in more than 50 samples were included. FDR cutoff values were estimated using the propr function *updateCutoffs*. We created a network representation of the resulting MSP pairs with proportionality values greater than 0.65.

### Random forest classification model to predict disease phenotype

We trained a random forest classifier with hyperparameters “bootstrap”: true, “ccp_alpha”: 0.0, “class_weight”: none, “criterion”: “criterion,” “max_depth”: none, “max_features”: “auto,” “max_leaf_nodes”: none, “max_samples”: none, “min_impurity_decrease”: 0.0, “min_samples_leaf”: 1, “min_samples_split”: 2, “min_weight_fraction_leaf”: 0.0, “n_estimators”: 500, “n_jobs”: –1, “oob_score”: false, “random_state”: 1, “verbose”: 0, “warm_start”: false to distinguish between equal numbers of disease and healthy controls for each disease data set that has a corresponding matched healthy control using the scikit-learn Python package (Pedregosa et al. 2011). First, the relative abundance data were standardized using the scikit-learn implementation of the *StandardScaler* function. Training and testing were performed on randomly selected samples split 70% and 30% of the full diseased data set, respectively, with a fixed random seed to ensure the reproducibility of the model. Hyperparameters were tuned using Python package “*Pycaret*” (Pol and Sawant 2021). Model performance was measured using AUROC scoring. Python implementation of the explainable AI algorithm SHAP was used to show the species contribution to disease classification (Lundberg and Lee 2017). The mean absolute SHAP score for each disease predictive model was determined using the sign of the Spearman's rank correlation between the feature value and the SHAP score. Positive values indicate that a higher relative abundance is more likely to classify the disease than in healthy samples. Negative values indicate that a lower relative abundance is more likely to classify the disease than in healthy samples.

### Data sets

The list of public data sets used in this study are available at https://www.microbiomeatlas.org, on the downloads page under “bioproj.csv” with relevant project accession code of the raw data and references. Additionally, these data sets were provided in Supplemental Table S1. In the case of the samples, their metadata were available (including age, gender, BMI, and geography); they are provided in the https://www.microbiomeatlas.org, download page under the “sampleID.csv.” The complete interactive MSP phylogenetic tree with effect size and western versus nonwestern annotations in Supplemental Figure S5 is accessible through the “iTOL” link (https://itol.embl.de/tree/130237251127435861638193829). The downloadable link for the genome-scale metabolic models (GEMs) linked to the MSPs is provided at https://www.microbiomeatlas.org, on the downloads page under “MSP_GEM_models.zip,” and the construction and details of the GEMs have been reported in our other paper (Bidkhori and Shoaie 2024).

### Software availability

The scripts for functional cluster characterization, SHAP calculation, plotting, and enrichment in disease/region are included in the Supplemental Code and are publicly available at GitHub (https://github.com/sysbiomelab/ATLAS). The R language computing program was used for most of the analysis (R Core Team 2022).

## Supplementary Material

Supplement 1

Supplement 2

Supplement 3

Supplement 4

Supplement 5

Supplement 6

Supplement 7

Supplement 8

Supplement 9

Supplement 10

Supplement 11

Supplement 12

Supplement 13

Supplement 14
