# Supplementary material for: Global compositional and functional states of the human gut microbiome in health and disease
Source: Genome Res. 2024 Jun;34(6):967–78. doi: 10.1101/gr.278637.123 (PMC11293553; doi:10.1101/gr.278637.123)
Supplement: Supplement 14 [file Supplemental_Material.pdf]

Supplemental Material

Global compositional and functional state of the human gut microbiome in health and disease

Sunjae Lee, Theo Portlock, Emmanuelle Le Chatelier,
Fernando Garcia-Guevara, Frederick Clasen,
Florian Plaza Onate, Nicolas Pons, Neelu Begum,
Azadeh Harzandi, Ceri Proffitt, Dorines Rosario,
Stefania Vaga, Junseok Park, Kalle von Feilitzen,
Fredric Johansson, Cheng Zhang, Lindsey A. Edwards,
Vincent Lombard, Franck Gauthier, Claire J. Steves,
David Gomez-Cabrero, Bernard Henrissat,
Doheon Lee, Lars Engstrand, Debbie L. Shawcross,
Gordon Proctor, Mathieu Almeida, Jens Nielsen
Adil Mardinoglu, David L. Moyes, Stanislav Dusko Ehrlich,
Mathias Uhlen, Saeed Shoaie

Table of contents

1 Supplemental Figures **2**

2 Supplemental Table Legends **10**

3 Supplemental Methods **11**

3.1 Metagenomics species pan-genome (MSP) creation 11

1 Supplemental Figures

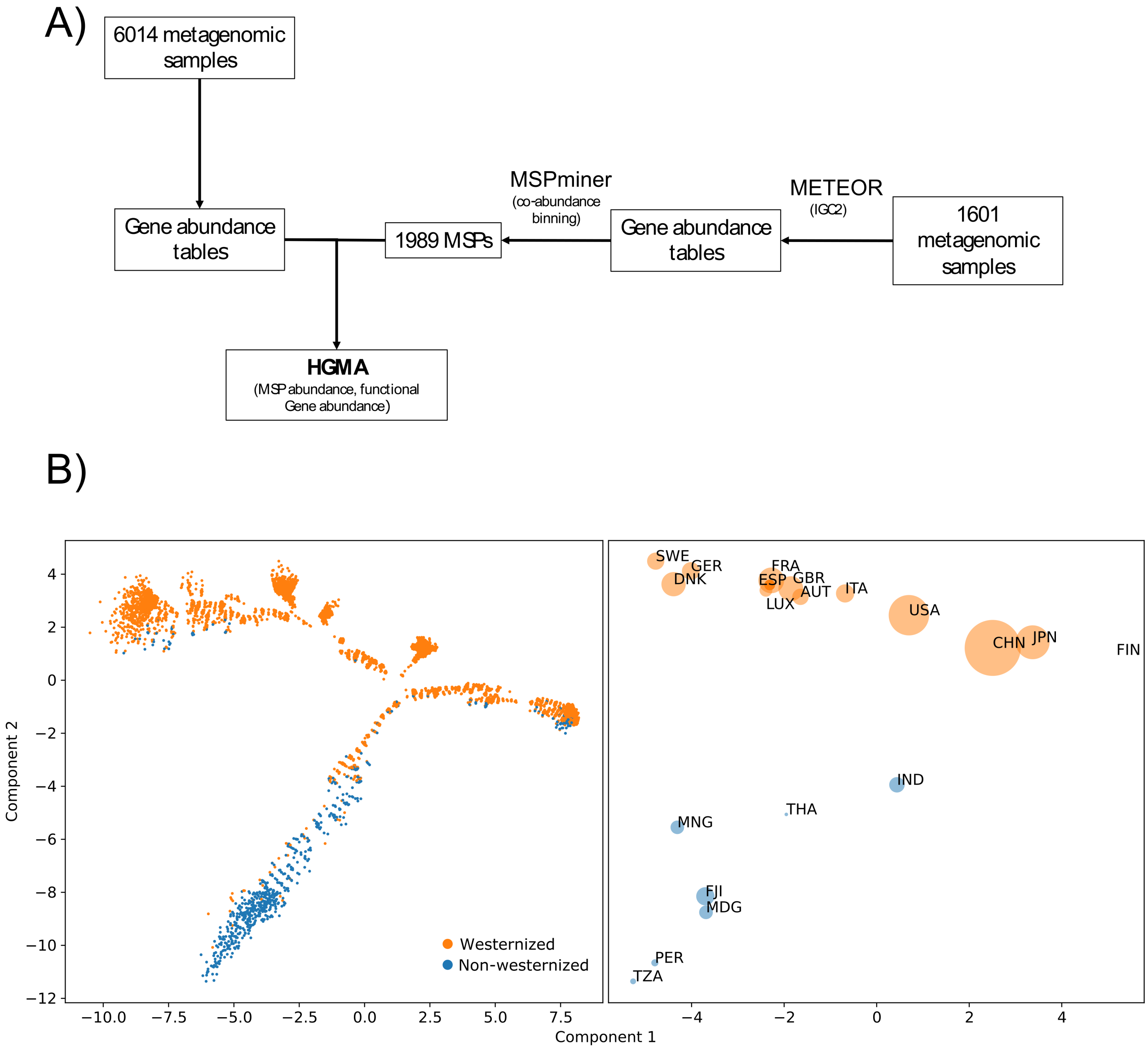

Supplemental Figure S1. **A**, Flow diagram depicting the workflow used to reconstruct MSPs and the Human Gut Microbiome Atlas (HGMA). **B**, Monocle ordination of the gut microbiome related to Figure 1E where nodes show country of origin.

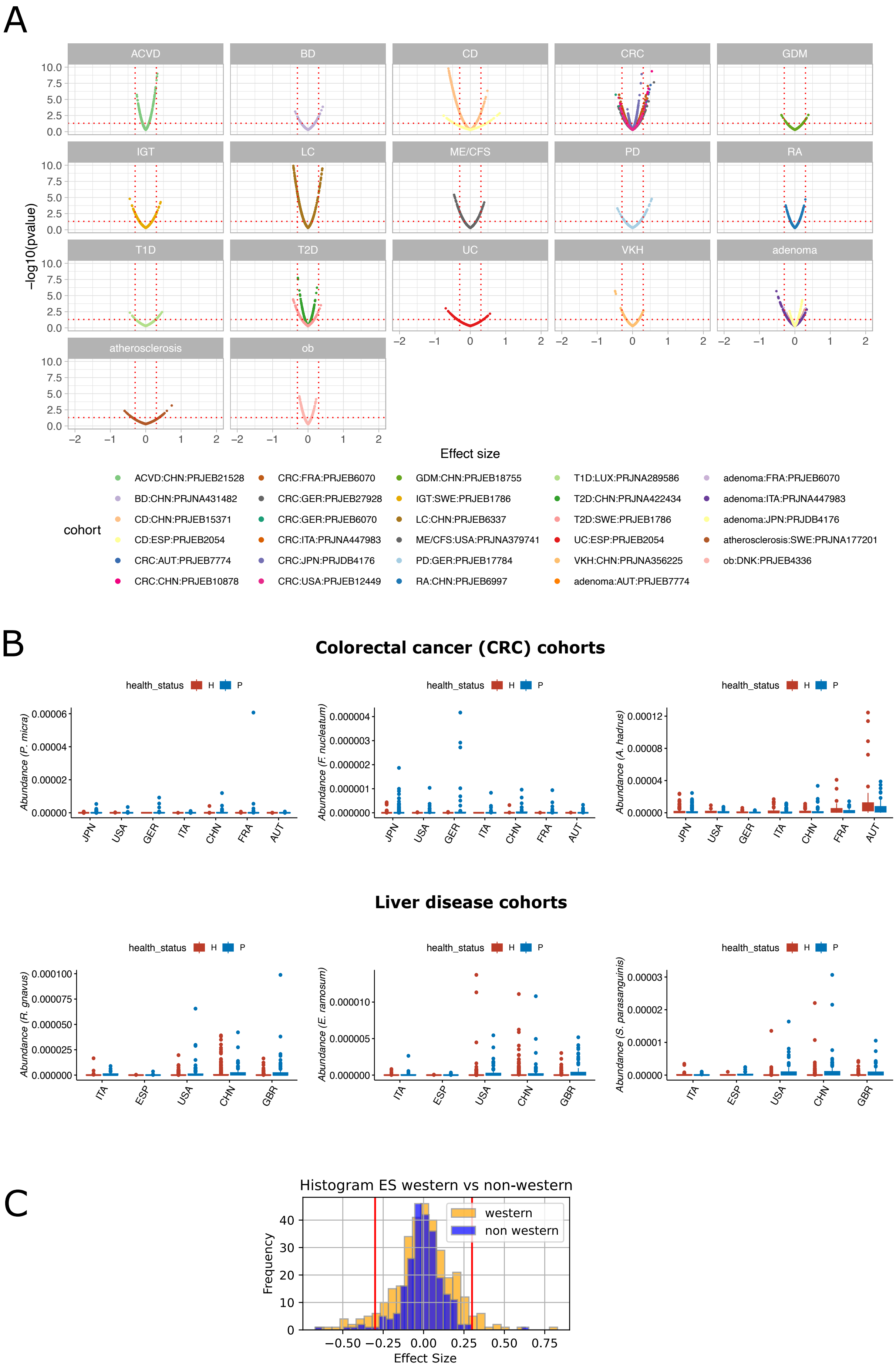
A
ACVD
BD
CD
CRC
GDM
IGT
LC
ME/CFS
PD
RA
T1D
T2D
UC
VKH
adenoma
atherosclerosis
ob
−log10(pvalue)
Effect size
cohort
ACVD:CHN:PRJEB21528
BD:CHN:PRJNA431482
CD:CHN:PRJEB15371
CD:ESP:PRJEB2054
CRC:AUT:PRJEB7774
CRC:CHN:PRJEB10878
CRC:FRA:PRJEB6070
CRC:GER:PRJEB27928
CRC:GER:PRJEB6070
CRC:ITA:PRJNA447983
CRC:JPN:PRJDB4176
CRC:USA:PRJEB12449
GDM:CHN:PRJEB18755
IGT:SWE:PRJEB1786
LC:CHN:PRJEB6337
ME/CFS:USA:PRJNA379741
PD:GER:PRJEB17784
RA:CHN:PRJEB6997
T1D:LUX:PRJNA289586
T2D:CHN:PRJNA422434
T2D:SWE:PRJEB1786
UC:ESP:PRJEB2054
VKH:CHN:PRJNA356225
adenoma:AUT:PRJEB7774
adenoma:FRA:PRJEB6070
adenoma:ITA:PRJNA447983
adenoma:JPN:PRJDB4176
atherosclerosis:SWE:PRJNA177201
ob:DNK:PRJEB4336
B
Colorectal cancer (CRC) cohorts
health_status H P
Abundance (P. micra)
Abundance (F. nucleatum)
Abundance (A. hadrus)
JPN USA GER ITA CHN FRA AUT
Liver disease cohorts
Abundance (R. gnavus)
Abundance (E. ramosum)
Abundance (S. parasanguinis)
ITA ESP USA CHN GBR
C
Histogram ES western vs non-western
western
non western
Frequency
Effect Size

Supplemental Figure S2. **A**, Volcano plots display Effect Size vs P-values across disease cohorts included in this study, each dot in the plot represents an MSP within a cohort. Red horizontal dotted line indicates P-value equal to 0.05, red vertical lines indicate Effect Size value equal to 0.3 **B**, Boxplots of selected species in colorectal cancer and liver disease cohorts to highlight the underlying data used for differential abundance analysis. **C**, Histogram with the Effect Size values across disease cohorts of the species enriched Western and Non-Western healthy groups. Red vertical lines indicate Effect Size absolute values of 0.3.

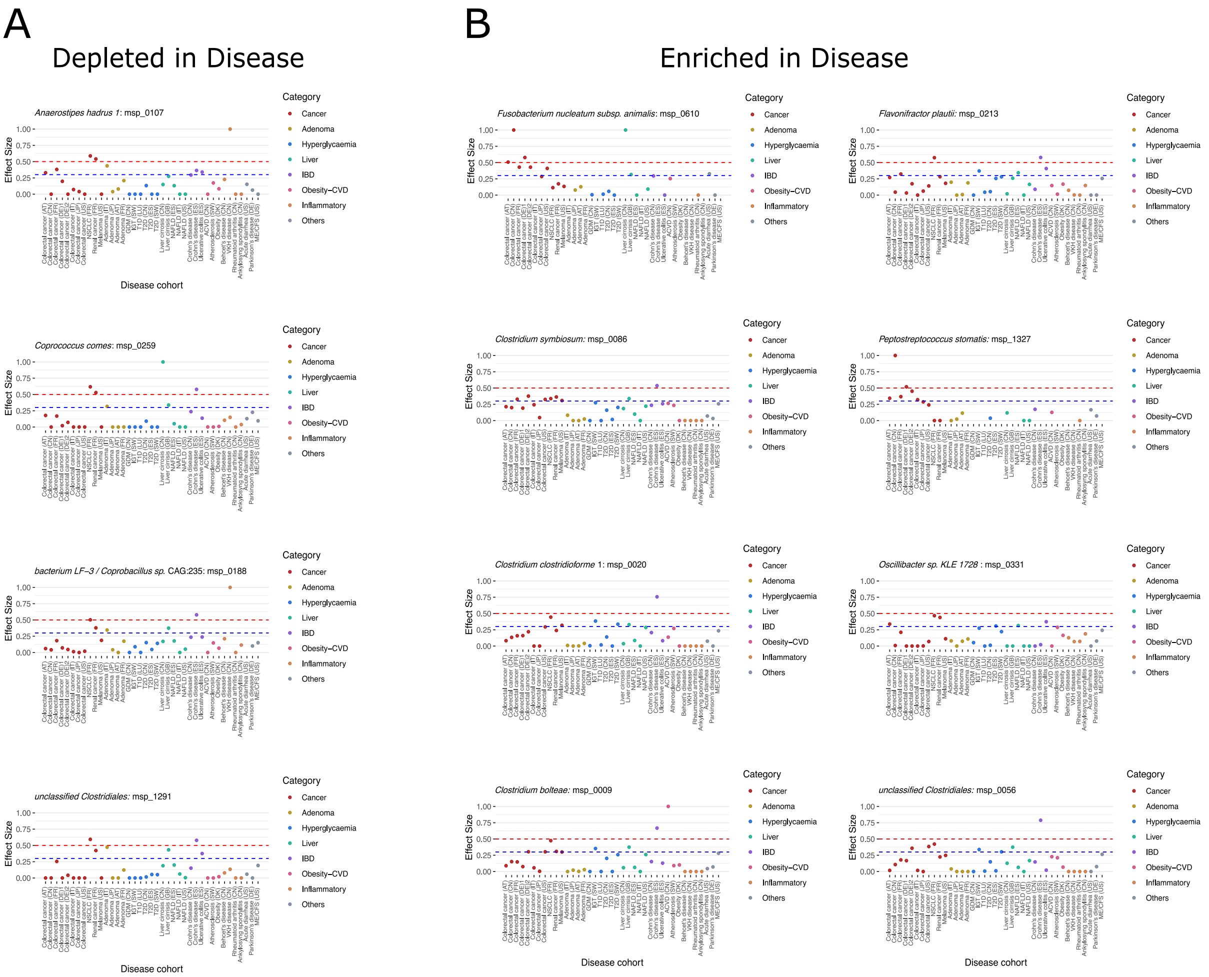

Supplemental Figure S3. Species found either **A** depleted or **B** enriched (Effect Size >0.3) in at least 6 different disease cohorts. The blue dotted line indicates Effect Size equals 0.3, the red dotted line indicates Effect Size equals 0.5, and each dot in the plot represents the Effect Size of the species within a disease cohort. Stars indicate cohorts where the Effect Size was above 0.3.

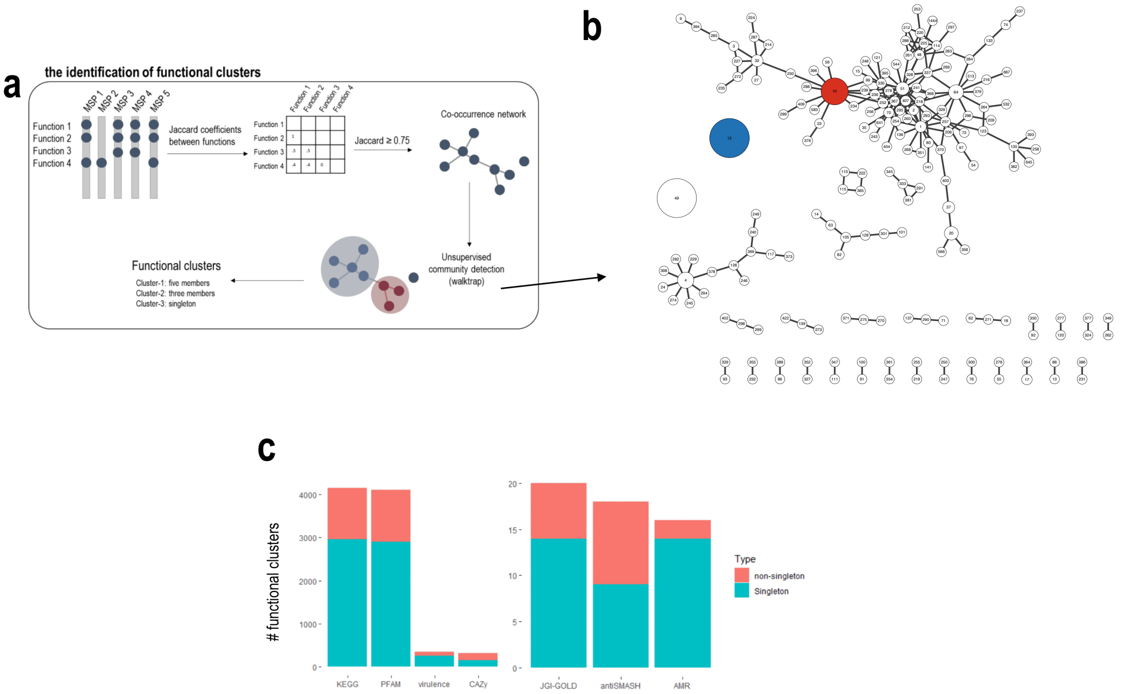

Supplemental Figure S4. Analysis of functional clusters. **A**, Overview of the identification of functional clusters. **B**, Community network detected from the co-occurrence network of functional annotations. All functional clusters were shown as nodes and significant cooccurrences of functional clusters across species were shown as edges in the network. "Comm-cluster" and "patho-cluster" were coloured blue and red, respectively. **C**, Number of functional clusters mapped with microbial function/phenotype databases and their singleton cluster fractions (singleton and non-singleton coloured green and red, respectively).

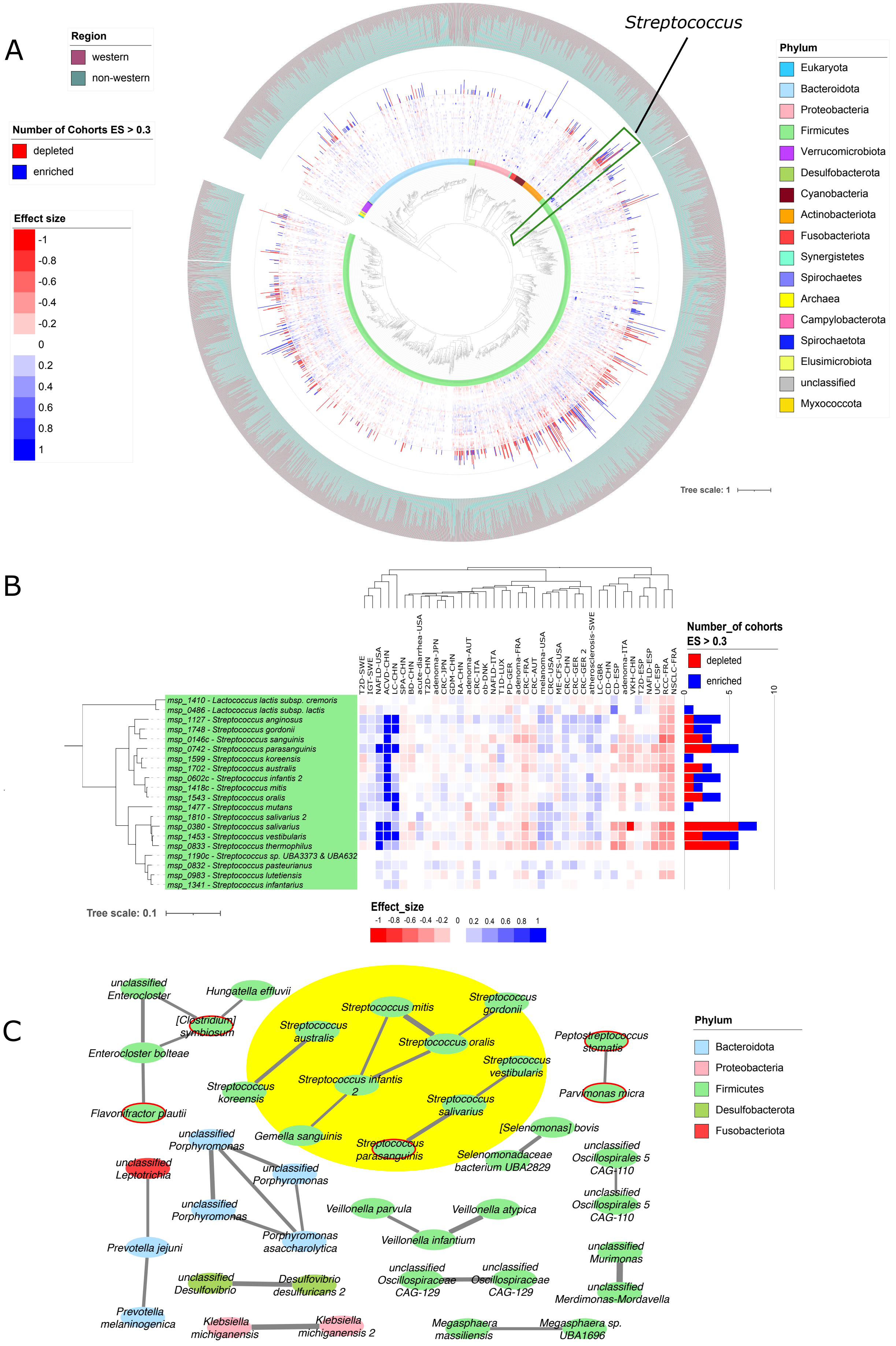
A
Streptococcus
Region
western
non-western
Number of Cohorts ES > 0.3
depleted
enriched
Effect size
-1
-0.8
-0.6
-0.4
-0.2
0
0.2
0.4
0.6
0.8
1
Phylum
Eukaryota
Bacteroidota
Proteobacteria
Firmicutes
Verrucomicrobiota
Desulfobacterota
Cyanobacteria
Actinobacteriota
Fusobacteriota
Synergistetes
Spirochaetes
Archaea
Campylobacterota
Spirochaetota
Elusimicrobiota
unclassified
Myxococcota
Tree scale: 1
B
T2D-SWE
IGT-SWE
NAFLD-USA
ACVD-CHN
LC-CHN
SPA-CHN
BD-CHN
acute-diarrhea-USA
T2D-CHN
adenoma-JPN
CRC-JPN
GDM-CHN
RA-CHN
adenoma-AUT
CRC-ITA
ob-DNK
NAFLD-ITA
T1D-LUX
PD-GER
adenoma-FRA
CRC-FRA
CRC-AUT
melanoma-USA
CRC-USA
ME-CFS-USA
CRC-CHN
CRC-GER
CRC-GER 2
atherosclerosis-SWE
LC-GBR
CD-CHN
CD-ESP
adenoma-ITA
VKH-CHN
T2D-ESP
NAFLD-ESP
UC-ESP
RCC-FRA
NSCLC-FRA
Number_of cohorts ES > 0.3
depleted
enriched
0
5
10
msp_1410 - Lactococcus lactis subsp. cremoris
msp_0486 - Lactococcus lactis subsp. lactis
msp_1127 - Streptococcus anginosus
msp_1748 - Streptococcus gordonii
msp_0146c - Streptococcus sanguinis
msp_0742 - Streptococcus parasanguinis
msp_1599 - Streptococcus koreensis
msp_1702 - Streptococcus australis
msp_0602c - Streptococcus infantis 2
msp_1418c - Streptococcus mitis
msp_1543 - Streptococcus oralis
msp_1477 - Streptococcus mutans
msp_1810 - Streptococcus salivarius 2
msp_0380 - Streptococcus salivarius
msp_1453 - Streptococcus vestibularis
msp_0833 - Streptococcus thermophilus
msp_1190c - Streptococcus sp. UBA3373 & UBA632
msp_0832 - Streptococcus pasteurianus
msp_0983 - Streptococcus lutetiensis
msp_1341 - Streptococcus infantarius
Tree scale: 0.1
Effect_size
-1 -0.8 -0.6 -0.4 -0.2 0 0.2 0.4 0.6 0.8 1
C
unclassified Enterocloster
Hungatella effluvii
[Clostridium] symbiosum
Enterocloster bolteae
Flavonifractor plautii
Streptococcus mitis
Streptococcus gordonii
Streptococcus australis
Streptococcus oralis
Streptococcus vestibularis
Streptococcus infantis 2
Streptococcus koreensis
Streptococcus salivarius
Gemella sanguinis
Streptococcus parasanguinis
Peptostreptococcus stomatis
Parvimonas micra
[Selenomonas] bovis
Selenomonadaceae bacterium UBA2829
unclassified Porphyromonas
unclassified Leptotrichia
unclassified Porphyromonas
unclassified Porphyromonas
Porphyromonas asaccharolytica
Prevotella jejuni
Prevotella melaninogenica
Veillonella parvula
Veillonella atypica
Veillonella infantium
unclassified Oscillospirales 5 CAG-110
unclassified Oscillospirales 5 CAG-110
unclassified Murimonas
unclassified Merdimonas-Mordavella
unclassified Desulfovibrio
Desulfovibrio desulfuricans 2
unclassified Oscillospiraceae CAG-129
unclassified Oscillospiraceae CAG-129
Klebsiella michiganensis
Klebsiella michiganensis 2
Megasphaera massiliensis
Megasphaera sp. UBA1696
Phylum
Bacteroidota
Proteobacteria
Firmicutes
Desulfobacterota
Fusobacteriota

Supplemental Figure S5. *Streptococcus* genus in the MSP phylogenetic tree. **A**, The Inner annotation indicates the species' phylum, and the heatmap displays the Effect Size for each MSP across all disease cohorts included in this study, The bar plots on the left side show the number of cohorts where an MSP's Effect Size was above 0.3. Red colour indicates depletion and blue colour, enrichment. Highlighted in green are the MSPs from the *Streptococcus* genus. The external barplot rings show the proportion of MSP abundance between western and non-western samples. The entire phylogenetic tree can be viewed interactively `https://itol.embl.de/tree/130237251127435861638193829`. **B**, Clustermap of the phylogenetic tree branch containing the MSPs from the *Streptococcus* genus and the estimated Effect Eize across all disease cohorts included in this study. The bar plots on the left side show the number of cohorts where an MSP's Effect Size was above 0.3. Red colour indicates depletion and blue colour, enrichment. The dendrogram on the top shows the hierarchical clustering result using the Ward method on the complete Effect Size matrix. **C**, Network representation of proportionality values between MSPs. Nodes represent MSPs and edges represent proportionality values above 0.65 between a pair of MSPs. Node fill colour indicates phylum, node edges in red highlight MSP with Effect Size >0.3 in more than two cohorts, and edge width is proportional to the proportionality value. The yellow oval in the background highlights the Clusters containing MSPs from the *Streptococcus* genus.

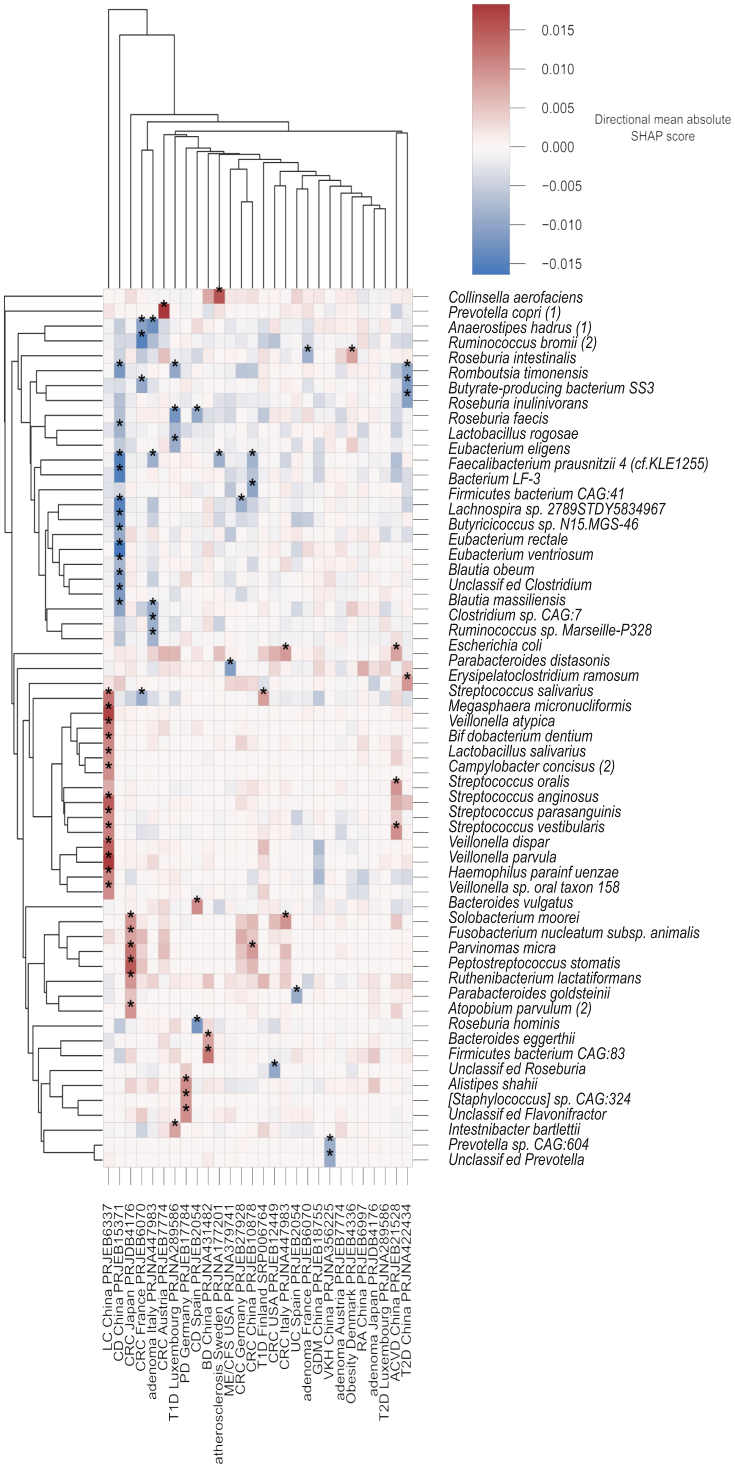

Supplemental Figure S6. Clustered heatmap related to Figure 4E with Euclidean dissimilarity clustering dendrograms included.

2 Supplemental Table Legends

Supplemental Table S1. Description of the Human Gut Microbiome Atlas (HGMA) datasets used in this study. We show the overall statistics of HGMA datasets, including the total number of samples, BioProject IDs, sequencing platform, geography, and reference.

Supplemental Table S2. List of region-enriched MSPs given as Z-score >2 and P-value <0.05, we identified 743 regionally enriched MSPs among healthy samples from 17 countries.

Supplemental Table S3. Comparison of species relative abundance between western and non-western groups.

Supplemental Table S4. Substrate information of CAZyme families commonly existed in human gut microbiome (Figure 1F).

Supplemental Table S5. Statistics of pan-metagenomics association study (pan-MGAS) results for 23 diseases. We performed pan-MGAS analysis and identified the enriched or depleted species in each cohort with effect sizes and P-values for each column. The negative sign in the effect size values indicated that the species was depleted in disease, positive effect size values, and species richness.

Supplemental Table S6. Statistics of common MSP enriched/depleted diseases. The table displays the MSPs with an effect size above 0.3 in the same direction in at least three different cohorts, along with the corresponding country, disease, and disease category.

Supplemental Table S7. Characteristics of 7,763 functional clusters. For a more detailed understanding, per functional cluster, we presented the size of the cluster, number of enriched species, enriched MSP identifiers, enriched species names, enriched KEGG modules (hypergeometric tests p <0.01), subsystems of enriched KEGG modules, KEGG orthologs terms, PFAM terms, virulence term, CAZyme terms, antimicrobial resistance (Mustard) terms, secondary metabolism (antiSMASH) terms, phenotype (JGI-GOLD) terms, product names of virulence terms, and all functional terms of a given cluster. **Supplemental Table S8.** Mean directional SHAP scores for each cohort with matched controls.

Supplemental Table S9. Statistical results of PERMANOVA of different batches of healthy samples of same country origins

Supplemental Table S10. Statistical results of PERMANOVA of confounder effects on healthy samples in different geographical datasets

Supplemental Table S11. Meta-analysis differential abundance testing between Healthy and Disease states controlling for age and gender using MMUPHin R package.

Supplemental Table S12. Summary of DNA extraction protocols of cohorts used in this study.

3 Supplemental Methods

3.1 Metagenomics species pan-genome (MSP) creation

To build MSP gene clusters clusters, MSPminer relies on the hypothesis initially introduced by Nielsen et al. that genes belonging to the same species should be co-abundant across multiple metagenomic samples. Although this assumption is mostly true for core genes, the co-abundance hypothesis has not been verified for genes shared by multiple species (horizontal transfers) or for genes subject to copy number variation. MSPminer introduces a new methodology that considers this limitation, capturing and classifying genes as part of the core genes (common to all strains of a given species) and accessory genes (specific to some strains only), with a higher clustering confidence for genes that are detected in multiple samples (*i.e.*, more than three samples).
